# Caldera-forming eruptions of mushy magma modulated by feedbacks between ascent rate, gas retention/loss and bubble/crystal framework interaction

**DOI:** 10.1038/s41598-019-52272-9

**Published:** 2019-11-01

**Authors:** Satoshi Okumura, Shanaka L. de Silva, Michihiko Nakamura, Osamu Sasaki

**Affiliations:** 1 Division of Earth and Planetary Materials Science, Department of Earth Science, Graduate School of Science, Tohoku University, Sendai, 980-8578 Japan; 2 College of Earth, Ocean, and Atmospheric Sciences, Oregon State University, Corvallis, OR97331-5503 USA; 3 Division of GeoEnvironmental Science, Department of Earth Science, Graduate School of Science, Tohoku University, Sendai, 980-8578 Japan

**Keywords:** Volcanology, Petrology

## Abstract

Caldera-forming eruptions of mushy silicic magma are among the most catastrophic natural events on Earth. In such magmas, crystals form an interlocking framework when their content reaches critical thresholds, resulting in the dramatic increase in viscous resistance to flow. Here, we propose a new mechanism for the ascent of mushy magma based on microstructural observations of crystal-rich silicic pumices and lavas from the Central Andes and decompression experiments. Microstructural data include spherical vesicles and jigsaw-puzzle association of broken crystals in pumices, whereas there is limited breakage of crystals in lavas. These observations insinuate that shearing of magma during ascent was limited. Decompression experiments reveal contrasting interaction between growing gas bubbles and the crystal framework in crystal-rich magma. Under slow decompression typical of effusive eruptions, gas extraction is promoted, whereas under rapid decompression, bubbles are retained and the crystal framework collapses. This feedback between decompression rate, retention of gas bubbles, and integrity of the crystal framework leads to strong non-linearity between magma decompression rate and eruption explosivity. We extend these findings to caldera-forming eruptions of crystal-rich magma where large overpressures are induced by caldera-collapse, resulting in magma plug-flow, rapid decompression facilitated by shear-localization at conduit margins, and explosive eruption.

## Introduction

Eruptions of mushy (crystal contents of >30 vol%) silicic magmas pose a challenge – they shouldn’t erupt because the viscosities of such magmas exceed theoretical “lock up” thresholds for magmas^1,2^. A theory of dike propagation to the surface and petrological estimates of pre-eruptive magma viscosity propose that the viscosity of eruptible magma in the reservoir must be less than ~10^6^ Pa s (refs^3–5^). Yet crystal-rich magmas with viscosities >10^6^ Pa s erupt effusively as lavas and explosively as fallout pyroclasts and pyroclastic density currents (ignimbrites), often associated with caldera-forming eruptions^6–8^. The challenge is exacerbated when the extreme events known as supereruptions are considered.

For caldera-forming eruptions with mushy silicic magmas, various different mechanisms for overcoming the rheological constraints have been proposed. Deep magma fragmentation under a high shear rate^8^ has been proposed to facilitate the rapid ascent of magma by reducing the viscosity through the transition from viscous fluid to gas-particle dispersion flow^9^. But this mechanism does not explain textural details in the eruptive products like high porosities, spherical vesicles and the jigsaw-puzzle association of broken crystals in pumices that are common features of explosive eruptions of crystal-rich silicic magma^10–12^. This is because a high porosity necessary for magma fragmentation cannot be achieved at high pressure (deeper in conduit or reservoir), and a high shear rate will result in the formation of sheared and elongated gas bubbles^13^. Another popular mechanism for overcoming the rheological constraints of mushy magma is thermal rejuvenation by intruding high temperature, volatile-rich magmas^6,14–16^. However, in this case the erupted magma should show clear textural evidence of thermal disequilibrium^17,18^. Yet there are multiple cases in which the crystal-rich magma erupts directly without any such evidence, as demonstrated in this study and ref.^8^.

An alternative condition that overcomes both these inconsistencies is when magma can ascend rapidly because of shear localization and viscous heating and consequent viscosity reduction of magma along conduit wall^19–21^. In the shear-localized zone, shear-induced bubble elongation, plastic deformation and breakage of crystals takes place^22–24^, whereas the magma body surrounded by the shear-localized zone experiences less simple shear and can retain gas bubbles and explosivity^25^. Still, effusive eruptions, thought to be caused by shear-induced outgassing during ascent^26,27^, cannot be explained by this model, because the formation of a shear-localized zone suppresses the outgassing of whole the magma^25^. These inconsistencies motivated us to look more closely at the questions surrounding the dynamics of caldera-forming eruptions of crystal-rich magma by investigating the microstructure of samples from ignimbrites and associated lavas from the Altiplano-Puna Volcanic Complex (APVC) of Central Andes, the products of one of the Earth’s most intense episodes of explosive eruption of crystal-rich magma^6,7^.

In this study, in addition to studying natural samples, we also performed decompression experiments of magma with 50 vol% crystals. On this basis we identify feedbacks between rate of magma ascent, retention of gas bubbles and integrity of the crystal framework that result in strong non-linearity between decompression rate and eruption explosivity. We propose that when crystal-rich magma ascends rapidly as a coherent mass (i.e., plug-type flow), formation, retention and growth of gas bubbles result in collapse of connected crystal networks, resulting in explosive eruptions. Conversely, slow decompression allows bubble transfer and outgassing through rigid crystal networks, eventually leading to effusive eruptions.

## Geological and Petrological Context

Many of the Earth’s largest ignimbrite flare-ups, like the one that took place during the Neogene in the Central Andes^28^, are characterized globally by ignimbrites and caldera complexes that are dominated by crystal-rich ignimbrites and lavas resulting from some of the Earth’s largest supereruptions^29^. Such ignimbrites are commonly referred to as “monotonous” because of the limited range in bulk chemistry of the dominant volume of erupted magma. In the Altiplano-Puna Volcanic Complex, covering the 20° to 24°S segment of the Central Andes, at least 15,000 km^3^ of magma was erupted during a ~10 Myr period, ~95% of which is classified as monotonous, crystal-rich, calc-alkaline dacite^7^. The lithological, volcanological, geochemical and petrological characteristics of the ignimbrites and lavas have been detailed in numerous studies over thirty years^30–34^. Bulk SiO_2_ contents range from 67 to 72 wt% and crystal contents between 30 and 55 vol%^7,31^. The magmas represented by both pumices and lavas were multiply saturated eutectoid compositions with five or six mineral phases (plagioclase + quartz + biotite + amphibole + Fe-Ti oxide ± sanidine) and calc-alkaline, high-K dacites to rhyodacites with high-Si rhyolite glass. Magma equilibration temperatures range from 700 °C to 850 °C and pre-eruptive storage pressures have been constrained to ~200 to 100 MPa^34–36^. This comprehensive foundation allows us to judiciously choose a small set of representative samples for the current study.

## Textural observations

The first phase of our investigation was to investigate bubble and phenocryst microstructure of pumices and lavas. Pumices from three ignimbrites and three samples of lavas representative of the compositional and textural ranges of the climactic ignimbrites and post-climactic lavas of the APVC were chosen (Table 1). Phenocryst contents of pumices and lavas measured by point counting under optical microscope are 42–51 and 35–50 vol%, respectively; total crystallinities in lavas are higher than 35–50 vol% due to the contribution of microlites. In pumices, microlites are absent, and the SiO_2_ contents in matrix glasses are >75 wt%. When we estimate the viscosity of the melt phase in magma based on the compilation of ref.^37^, we obtain melt viscosities of 10^5^ to 10^6^ Pa s. With phenocryst contents of 42–51 vol%, bulk magma viscosities are estimated to be 10^6^–10^8^ Pa s using the method of ref.^37^. For the lavas, the crystallinities and bulk SiO_2_ contents show similar ranges to the pumices, but a small amount of microlites are common, which can be formed through the decompression and dehydration during magma ascent^38^. Thus, the lavas are expected to have bulk viscosities exceeding 10^6^–10^8^ Pa s. The range of magma viscosities studied, is therefore at or beyond the threshold of magma eruptibility (~10^6^ Pa s) estimated from viscosities of natural erupted magmas and predicted by theoretical dike propagation^3–5^. Gas bubbles in magma can decrease the viscosity, but the degree of viscosity reduction at ~50 vol% crystals is not high, and is one order of magnitude at most^39–41^. On the other hand, the impact of gas bubbles increases with crystallinities and a small amount of gas bubbles can cause four orders of magnitude reduction at 70 vol% crystals^41^.

**Table 1:** Sample information, magma temperature, phenocryst content and porosity.

Sample	Locality	Rock type	Temperature^a^	Phenocryst content^a,b^	Porosity^c^
			(°C)	(vol%)	(vol%)
07BOL23PD	Puripica Chico ignimbrite, Bolivia	Dacite pumice	813 ± 25	51	51
07BOL011	Guacha ignimbrite, Bolivia	Dacite pumice	835 ± 25	46	41
83019	Puripicar ignimbrite, Chile	Dacite pumice	720 ± 17	42	69
07CB012A	Cerro Blanco, Argentina	Rhyolite lava	737 ± 15	35	43
09005CT	Cerro Chao, Chile	Dacite lava	809 ± 19	50	18
09011CT	Tocorpuri, Chile	Rhyolite lava	832 ± 8	47	29

^a^Temperature and phenocryst content are from refs^32,74,75^.

^b^The phenocryst content represents the volume fraction of phenocrysts on a pore-free basis.

^c^The porosity is the volume fraction of pores in a bulk sample, estimated from bulk density and by assuming the solid density of 2700 kg m^−3^.

**Figure 1:**
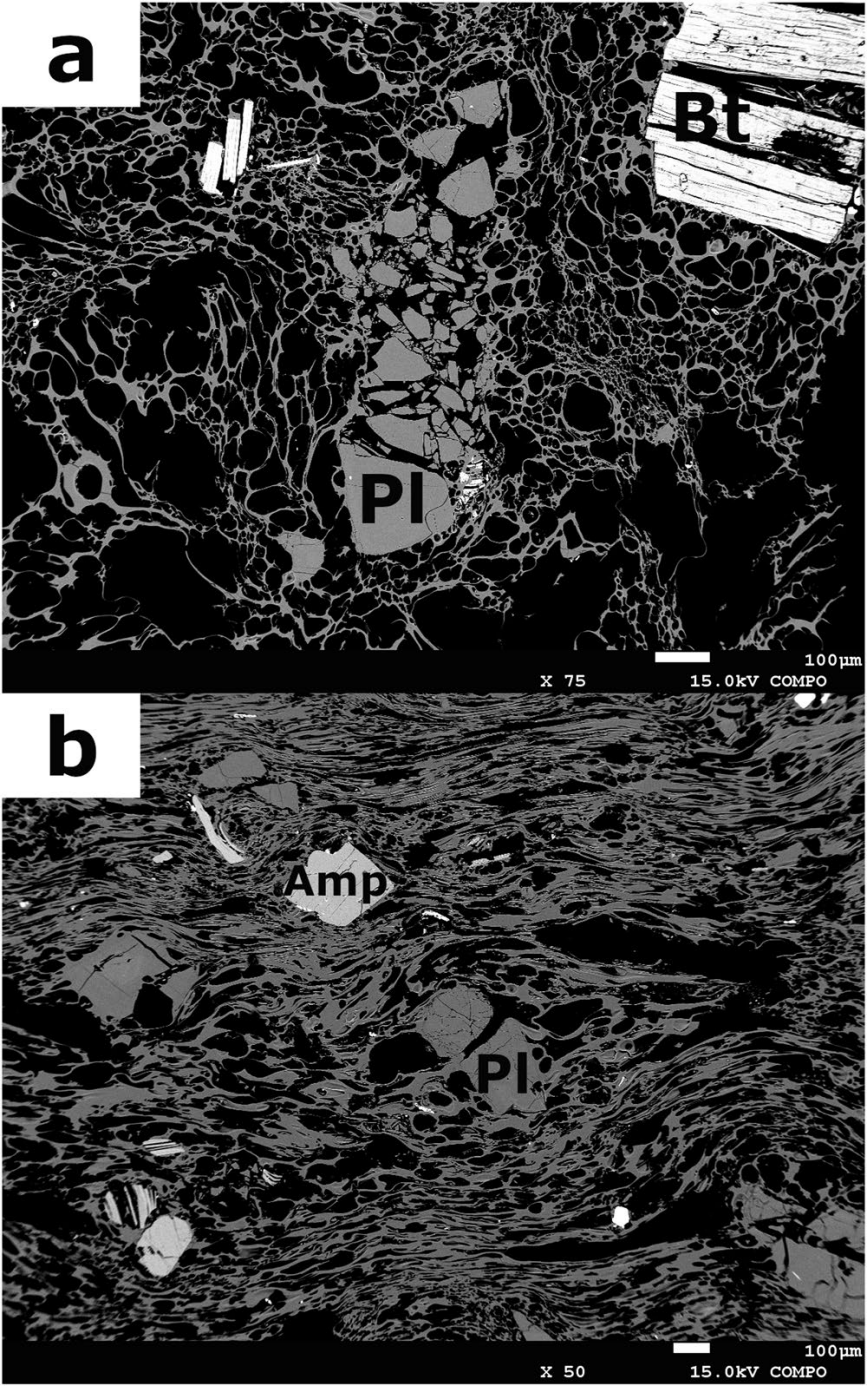
Backscattered electron images of pumices with spherical and sheared bubbles (**a**, 83019; **b**, 07BOL23PD). Black portions are bubbles; grey portions are groundmass and plagioclase. White to light grey particles represent mafic minerals. The image of a also shows broken phenocrysts in the centre. Pl, Bt and Amp represent mineral phases of plagioclase, biotite and amphibole, respectively.

**Figure 2:**
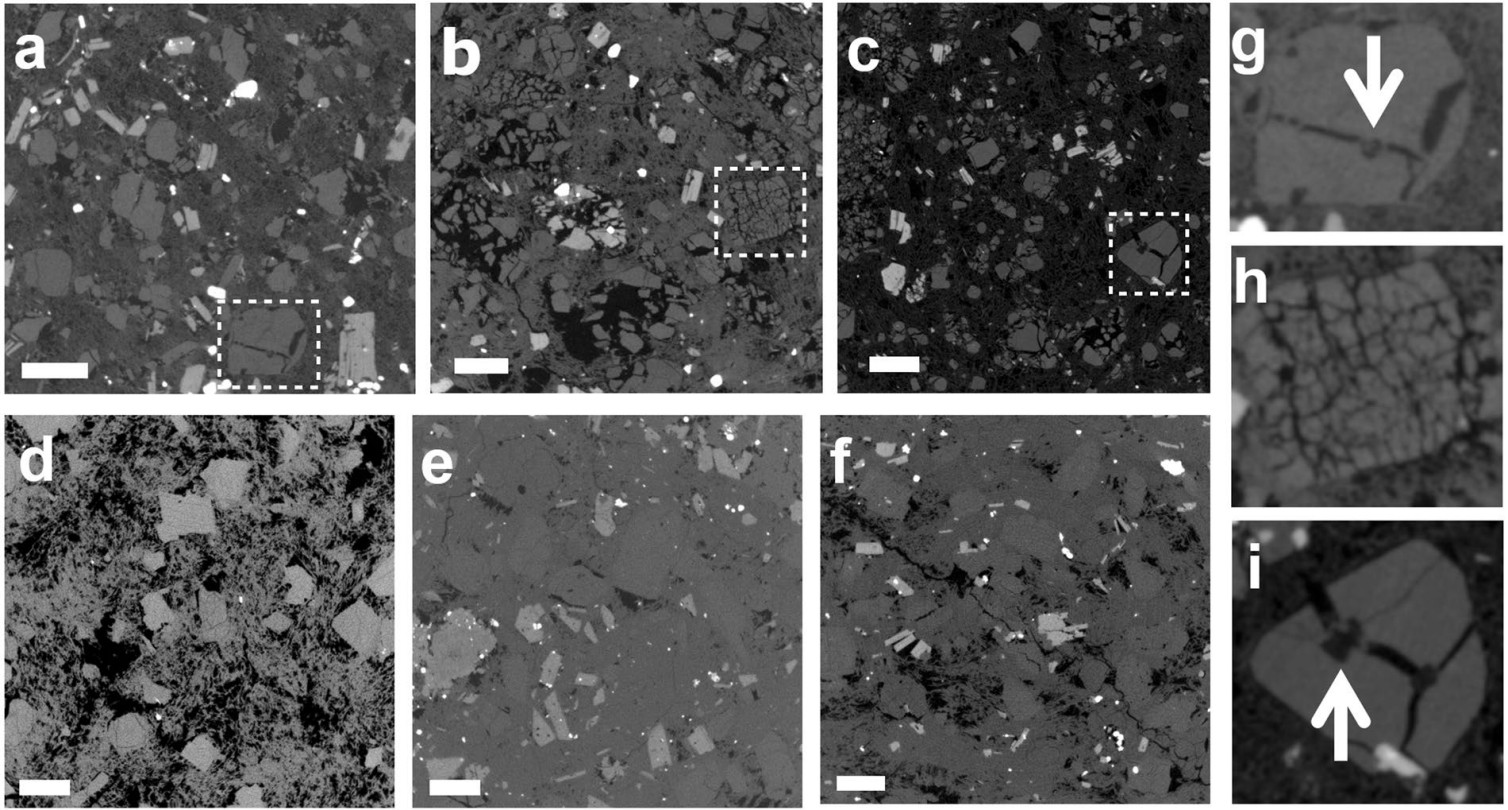
Typical CT images of pumices (**a**, 07BOL23PD; **b**, 07BOL011; **c**, 83019) and lavas (**d**, 07CB012A; **e**, 09005CT; **f**, 09011CT). The images of (**g**–**i**) show broken phenocrysts, which correspond to the areas enclosed by white dotted lines in (**a**–**c**), respectively. The black portions represent pores/vesicles. The grey portion corresponds to felsic minerals and groundmass. The light grey and white particles are mafic minerals. Foamed melt inclusions are found along fractures inside the phenocrysts in (**g**,**i**) (white arrows). (**h**) Represents an example of phenocryst fragmentation without foamed melt inclusion. The white bars represent 1 mm scale.

Bulk porosities of pumices and lavas are 41–69 and 18–43 vol%, respectively (Table 1). Bubbles with ~10 to ~100 μm in diameter are almost spherical in pumices (Fig. 1a). Images of pumices and lavas obtained by micro-X ray computed tomography (CT) (see Methods) also reveal important differences (Fig. 2). In pumices, many crystals are broken and arranged in a jigsaw puzzle fashion (see also Fig. 1a). The intensity of breakage ranges widely from one grain into many pieces to fine fragments (Fig. 2a–c). Broken phenocrysts include foamed melt inclusions (MIs) (Fig. 2g,i), suggesting rapid decompression of magma and foaming of MIs^10,11,42^. However, foamed MIs are not found in the finely broken phenocrysts (Fig. 2h). Broken crystals show little or no relative movement of fragments after their breakage, indicating limited flow after breakage of the phenocrysts. This fragmentation could be syn-eruptive or through stress accumulation along crystal network during post-eruptive processes such as welding and compaction of large ignimbrite deposits^12^. For this, a crystal network that supports the force applied to the system is necessary, and a bulk volume crystallinity (the ratio of crystal volume fraction to total rock volume) of ~25 vol% is large enough for the formation of such a connected crystal network^43–45^. However, the pumices from Puripicar Ignimbrite studied here have high vesicularities and lower bulk volume crystallinity (13 vol%, calculated based on bulk porosity of 69 vol% and pore free-basis phenocryst content of 42 vol% in Table 1) than this percolation threshold. Additionally, most of phenocrysts in pumices studied show fragmentation, even though some of phenocrysts are not involved in force chain. Hence, the phenocrysts were not fragmented by post-eruptive processes and we conclude that phenocryst fragmentation is the result of stress accumulation between the phenocrysts during magma ascent and eruption due to a high decompression rate and high viscosity of the silicic melts. In contrast, lava samples that we studied do not reveal this type of crystal breakage, implying suppression of MI foaming and lower stress accumulation in effusive eruptions due to lower rate of magma decompression and limited shearing during flow.

Post-fragmentation evolution of the gas bubbles in silicic magmas with high viscosity is not significant^46,47^. Therefore, spherical vesicles and high to moderate porosities in pumices, based on the classification of ref.^48^, imply that the magmas were not strongly deformed under a high shear flow^49^ and decompressed to low pressures just before magma fragmentation; sheared pumices that are found as a minor component in the ignimbrite (Fig. 1b) correspond to the magma that was significantly sheared in the conduit – a thin annulus surrounding the core plug flow.

In contrast, the porosities of lavas are lower than those of pumices (Table 1). Since hydrous minerals appear to be in equilibrium in the lavas as well as in the pumices, water contents in the effused magmas should have been high enough to cause magma vesiculation. Thus, we conclude that the lower porosity must result from outgassing during magma ascent and emplacement. However, the mechanism of outgassing from crystal-rich magma is unclear. It has been proposed that shear deformation enhances outgassing^22,26,27^, but this cannot explain efficient outgassing from crystal-rich magma, because the shear is localized, resulting in the suppression of large-scale deformation of crystal-rich magmas^50,51^. We have investigated this experimentally.

## Experiments

We experimentally simulated the formation of gas bubbles under decompression and investigated the evolution of microstructure using micro-X ray CT (see Methods). The decompression experiments were performed under similar temperature condition (i.e., 800 °C) with those of magmas studied above, for rhyolitic melt with 50 vol% corundum crystals. At 50 vol% crystals, roughly equant corundum crystals form the framework, resulting in high crystal connectivity, where the connectivity of the crystals (Φ_c_) is defined as the ratio of the volume of crystals belonging to the largest crystal network to the total crystal volume. This means that a substantial number of crystals are connected when Φ_c_ is high. To describe the degree of bubble connection, we use the connectivity of bubbles (Φ_b_), which is defined as the ratio of the volume of bubbles belonging to the largest bubble network to the total bubble volume.

We performed two series of decompression experiments. In the first series, EX-I, we rapidly decompressed the rhyolitic melts with 50 vol% crystals from 100 to 40, 20, 15 and 10 MPa, and decompressed to 20 MPa at a rate of 8 MPa h^−1^. In the second series, EX-II, rhyolitic glass with 50 vol% crystals was sandwiched between crystal-free rhyolite glasses (reference material JR-1 from the Geological Survey of Japan) in the capsule. These hydrous melts were subjected to a continuous decompression to 20 MPa at rates of 8, 80, 320 and 3200 MPa h^−1^ and rapid decompression (~28800 MPa h^−1^). The decompression rate of 8 MPa h^−1^ represents rates during lava effusion^52,53^. During explosive eruption, decompression rates estimated from various techniques show wide ranges^54^, but our experimental rates of 320 and 3200 MPa h^−1^ and rapid decompression (~28800 MPa h^−1^) cover those of the explosive eruptions. The experimental condition and results are summarized in Table S1.

**Figure 3:**
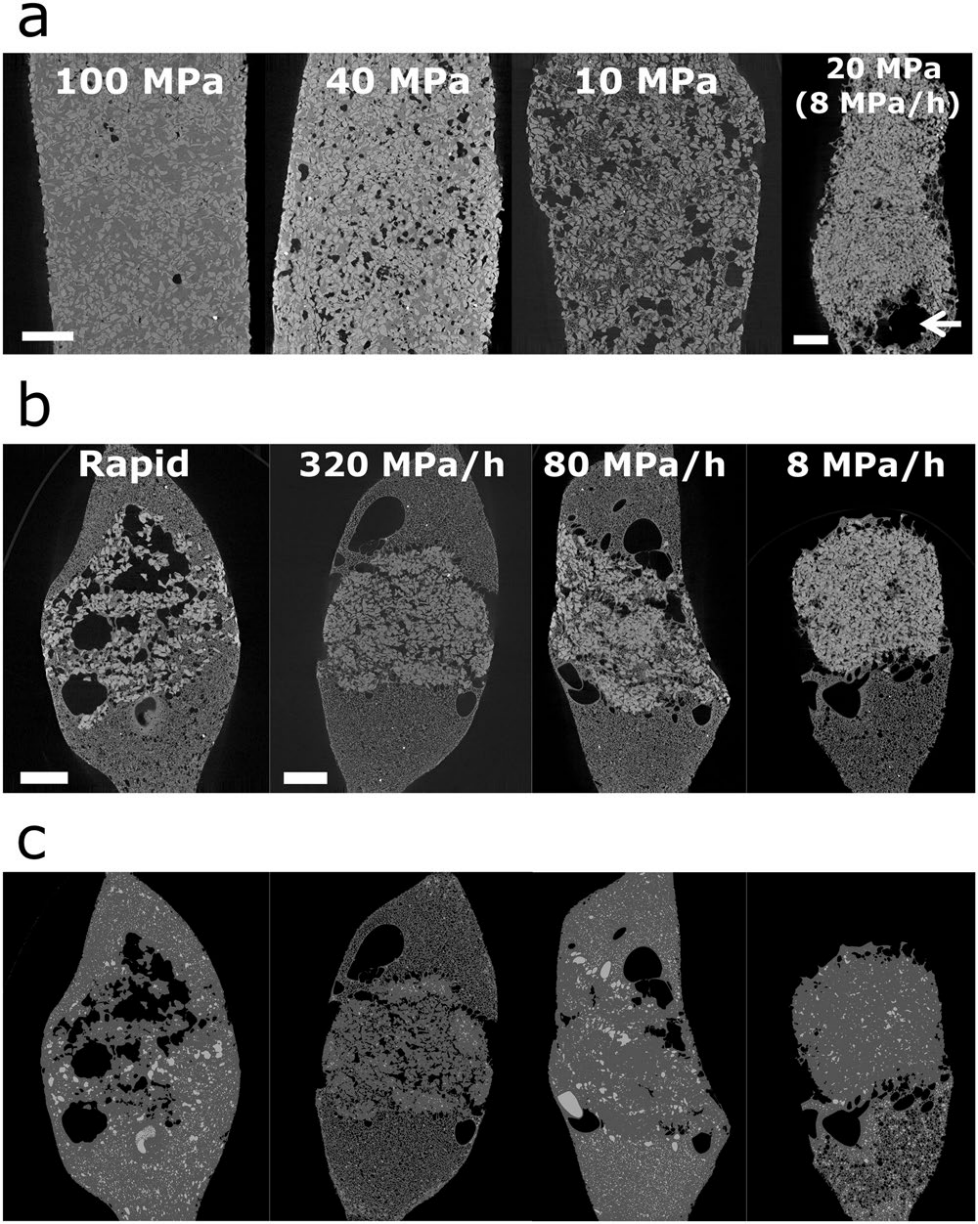
Slice CT images of (**a**) EX-I, (**b**) EX-II products and (**c**) images highlighting connected and isolated gas bubbles in EX-II products. In (**a**) and (**b**), black and grey portions in the samples represent gas bubbles and rhyolitic glasses. Light grey particles are Al_2_O_3_ crystals. White bars indicate 1 mm long (100, 40 and 10 MPa, and 320, 80 and 8 MPa h^−1^ have the same scales). In (**a**) EX-I with 8 MPa h^−1^, a large pore is found in the bottom, which was caused by gas extraction (white arrow). In (**b**) EX-II, gas extractions from crystal-rich (central) to crystal-free (upper and lower) zones are found in all runs, while the vesiculation also lubricates the crystal-rich zone (see also Fig. 4). Owing to its fragility, the upper portion of the sample with 8 MPa h^−1^ was broken. In (**c**), three-dimensionally interconnected bubbles in the samples correspond to black portions, and light grey portions in the sample represent isolated gas bubbles. Interconnected bubbles exist in crystal-rich zone at decompression rates more than 320 MPa h^−1^, while tiny isolated gas bubbles are dominant at decompression rates of 80 and 8 MPa h^−1^ as a result from gas extraction and compaction.

**Figure 4:**
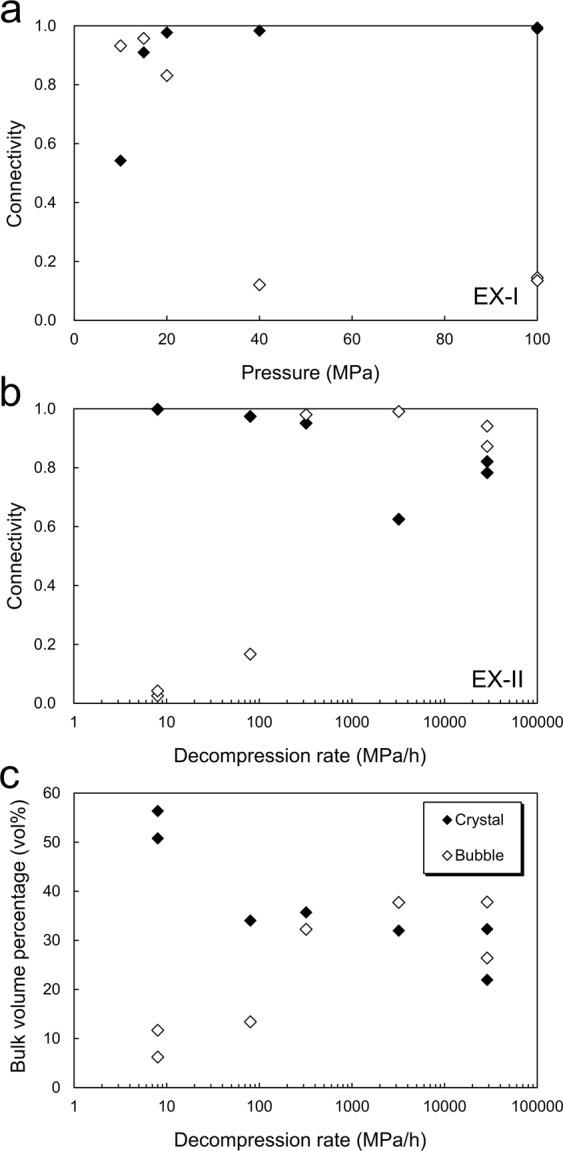
Evolution of bubble and crystal connectivities (Φ_b_ and Φ_c_), bulk volume crystallinity and vesicularity. (**a**) The bubble and crystal connectivities obtained from the EX-I series. (**b**) The bubble and crystal connectivities obtained from the EX-II series. The bubble connectivities show rapid increase between 80 and 320 MPa h^−1^. The crystal connectivity decreases with an increase in decompression rate. (**c**) Bulk volume crystallinities and vesicularities of crystal-rich zone from the EX-II series. Bulk volume crystallinity and vesicularity represent the percentages of crystals and gas bubbles in total volume. The bulk volume crystallinity decreases with an increase in decompression rate, while the vesicularity increases.

Examination of the microstructure of run products (Fig. 3) reveals that in the EX-I series of experiments, whereas the amount of gas bubbles and Φ_b_ increases with decompression (Fig. 4a and Table S1), extraction of gas bubbles is clear only at slow decompression (8 MPa h^−1^) that correspond to effusive eruptions (Fig. 3a). We also found a clear reduction in Φ_c_ due to vesiculation in the EX-I samples with 50 vol% crystal (Fig. 4a and Table S1). Before decompression, bulk porosities are <3 vol% and Φ_c_ is ~1. The value of Φ_c_ starts to decrease at a pressure of 20 MPa and reaches 0.54 at 10 MPa. Theoretical bulk porosities that are calculated based on water solubility law and using the equation of the state of an ideal gas are roughly consistent with the measured porosity (Table S1). This indicates that vesiculation was in equilibrium and controlled Φ_c_ in crystal-rich magma under rapid decompression.

In the EX-II series, the extraction of gas bubbles from crystal-rich magma was highlighted. The CT images demonstrate that gas bubbles expand in crystal-rich zone, resulting in the collapse of crystal framework under rapid decompression (Fig. 3b), whereas under rates of 8 and 80 MPa h^−1^ large gas bubbles are transferred from the crystal-rich zone to the crystal-free zone and only isolated tiny bubbles are left in crystal-rich zone (Fig. 3b,c). Quantitative data also exhibit that under decompression rates >320 MPa h^−1^, interconnected gas bubbles exist in crystal-rich zone, i.e., high Φ_b,_ and Φ_c_ is low (Fig. 4b), while the interconnected bubbles disappear, i.e., low Φ_b_, and Φ_c_ remains high at a decompression rate less than 80 MPa h^−1^ (Fig. 4b). Furthermore, rapid decompression causes a decrease in bulk volume crystallinity down to 22 vol% because of bubble formation and growth, whereas crystallinity remains ~50 vol% at a decompression rate of 8 MPa h^−1^ (Fig. 4c). The vesicularities are also high and low at high and low decompression rate, respectively (Fig. 4c). All of these observations indicate that decompression rate controls whether crystal-rich magma retains gas bubbles and the crystal framework collapses or whether the gas extracts from a rigid crystal framework. Only a small amount of microlites formed even at slow decompression (8 MPa h^−1^) (Fig. S1), suggesting that the effect of decompression-induced crystallization on gas extraction is negligible under the timescale of the experiments.

We emphasize that the extraction of gas bubbles did not occur after the growth of gas bubbles and the collapse of the crystal framework in the crystal-rich zone. Instead, the extraction was continuous under slow decompression while maintaining the crystal framework. The experiments in which the sample was decompressed to 20–40 MPa with a decompression rate of 8 MPa h^−1^ show that Φ_b_ and Φ_c_ are almost constant, i.e., 0.03–0.14 and 0.99–1, respectively (Table S1).

## Discussion

### Interpretation of the experimental results

We posit that the contrasting interaction between gas bubbles and the crystal framework is controlled by timescales of the increase in overpressure (*τ*_p_) and gas extraction (*τ*_ex_). During decompression, gas bubbles form in the crystal-rich zone as well as in the crystal-free zone. In the crystal-rich zone, the growth of gas bubbles is suppressed by the crystal framework, resulting in an increase in overpressure. When the strength of the crystal framework is high, resulting in a stable framework, the gas escapes to the crystal-free zone because of the resulting pressure gradient, as observed in the low to medium decompression-rate experiments (8–320 MPa h^−1^ in Fig. 3b,c). In contrast, once the overpressure caused by growing gas bubbles becomes greater than the strength of the framework, the bubbles expand in the crystal-rich zone and disrupt the framework (Rapid and 320 MPa h^−1^ in Fig. 3b,c).

**Figure 5:**
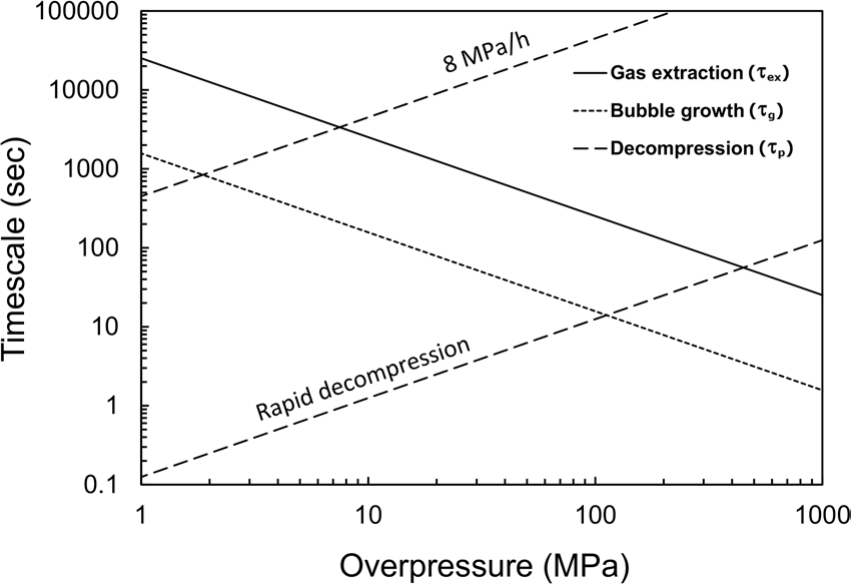
The theoretical estimate on the timescales of gas extraction (*τ*_ex_) and bubble growth (*τ*_g_) in crystal-rich magma. The time-overpressure relationships (*τ*_p_) during decompression with a rate of 8 MPa h^−1^ and rapid decompression (28800 MPa h^−1^) are also shown, as the increase in pressure is caused by decompression.

To understand this process quantitatively, we estimate the timescale for gas extraction (*τ*_ex_) from the crystal-rich zone to crystal-free zone based on Darcy’s law (see Methods). Figure 5 indicates that *τ*_ex_ decreases with an increase in the overpressure. However, overpressure yielding in the crystal-rich zone increases linearly with time (*τ*_p_) under the constant decompression rate when it is not reduced through gas extraction and bubble growth. With increasing overpressure, *τ*_ex_ approaches *τ*_p_. Here, the overpressure at which *τ*_ex_ is comparable with *τ*_p_ depends on decompression rate (Fig. 5). At a high decompression rate (28800 MPa h^−1^), large overpressures (~100 MPa) develop in crystal-rich zone; hence, when the internal pressure overcomes the strength of crystal framework, the crystal framework is disrupted, resulting in a dramatic decrease in magma viscosity. Thus, the overpressure is reduced by the growth of gas bubbles rather than gas being extracted to the crystal-free zone, because the timescale of bubble growth (*τ*_g_) (see Methods) is shorter than *τ*_ex_ (Fig. 5). On the other hand, at low decompression rates, *τ*_p_ approaches *τ*_ex_ at the overpressure of < 10 MPa (Fig. 5). In this case, the gas extraction reduces overpressure without the growth of gas bubbles in crystal-rich zone.

In this study, we used crystals with isotropic shape; however, the strength of crystal framework seems to depend on the crystal shape^45^. Therefore, magma hosting a crystal framework consisting of dominantly elongated crystals, as in the natural samples, may be more rigid, thereby enhancing the escape of gas. Additionally, the gas extraction may start at lower crystallinity when crystals have elongated shape, because crystal networks can form at lower crystallinity^55,56^.

### Eruptions of crystal-rich magma modulated by feedbacks between magma decompression rate, gas bubble retention and integrity of the crystal network

The microstructural features that we have observed in our sample suite and experiments are reconciled if crystal-rich magma ascends within an annular shear-localized and less viscous zone^19,20,25^. Once the magma starts to ascend to the surface, the decompression rate is a key factor in controlling the style of the eruption. Our new decompression experiments demonstrated that in rapidly decompressing magma, the formation of gas bubbles induces a reduction in crystal connectivity. When this happens in nature, gas bubbles cannot escape from the ascending magma column, increasing pressure, disrupting the crystal framework, and finally causing magma fragmentation and explosive eruption. In contrast, when the decompression rate is low, gas bubbles migrate out of the magma along the pressure gradient without the collapse of the crystal framework. In nature, this migration cannot cause outgassing throughout the magma body in a volcanic conduit, instead the extracted gas bubbles form a permeable pathway, resulting in efficient gas loss via the pathway, which promotes effusive activity.

A similar gas extraction process in crystal-rich magma has been proposed under shear deformation^26,41^. In this process, shear stress is supported by the rigid crystal framework, causing a pressure gradient in the magma and the resulting gas extraction. However, our observations suggest that the transport of crystal-rich magma is by plug flow. In this scenario, the core of the magmatic column, i.e., the plug, ascends whilst undergoing a smaller degree of shear^50,51^. The magma in the plug is not sheared but often displays low vesicularity in lava samples, which probably results from gas extraction due to decompression-induced vesiculation in crystal-rich magma. Repeated shear-induced outgassing, fracturing and welding processes^25,57^ may also explain the low vesicularity in the lavas; however, if so, shear-induced crystal breakage and subsequent reduction of crystal size would be expected in the lavas^23^, but we do not find this.

### New insights for catastrophic caldera-forming supereruptions

Our textural and experimental insights provide new insights into the ascent of mushy magma during supereruptions such as those from which our natural samples were collected. In a simple sense, magma decompression rate depends on the overpressure in the reservoir^58^; if the overpressure is small, magma flux to the surface is low and hence the magma experiences dehydration, outgassing and crystallization during its ascent, resulting in the increase of magma viscosity, the loss of explosivity, and finally lava effusion^58,59^. Conversely, large overpressures in the reservoir cause rapid ascent and decompression of crystal-rich magma without the loss of explosivity (outgassing). This relationship between magma flux and overpressure also depends on other parameters such as initial volatile contents^60^ and evolution and dynamics of the magma reservoir^61,62^. However, recent work has emphasized that the evolution of overpressure depends critically on the size of the system and in particular by the thermomechanics of the country rock surrounding the pre-eruptive magma reservoir^4,8,63,64^ and two pathways for reservoir overpressure evolution leading to eruption initiation have been suggested. For small “cold” magma reservoirs <100 km^3^, internal processes related to second boiling and recharge may dominate to produce an internal overpressure that drives the eruption by exceeding the tensile failure of the country rock. In these cases, caldera collapse occurs after an underpressure condition is achieved following a certain level of magma withdrawal from a reservoir. For larger (supervolcanic) systems, like those that we have sampled here, ductile wall rocks to the magma reservoir modulate internal pressure development, which could even lead to underpressure in the magma reservoir without magma withdrawal^64^. In these largest eruptions, roof subsidence along outward dipping faults initiates the eruption, subsidence and eruption are coupled from the outset, and subsidence of the roof into the reservoir maintains high excess pressure during almost the entire eruption^63,65–67^.

**Figure 6:**
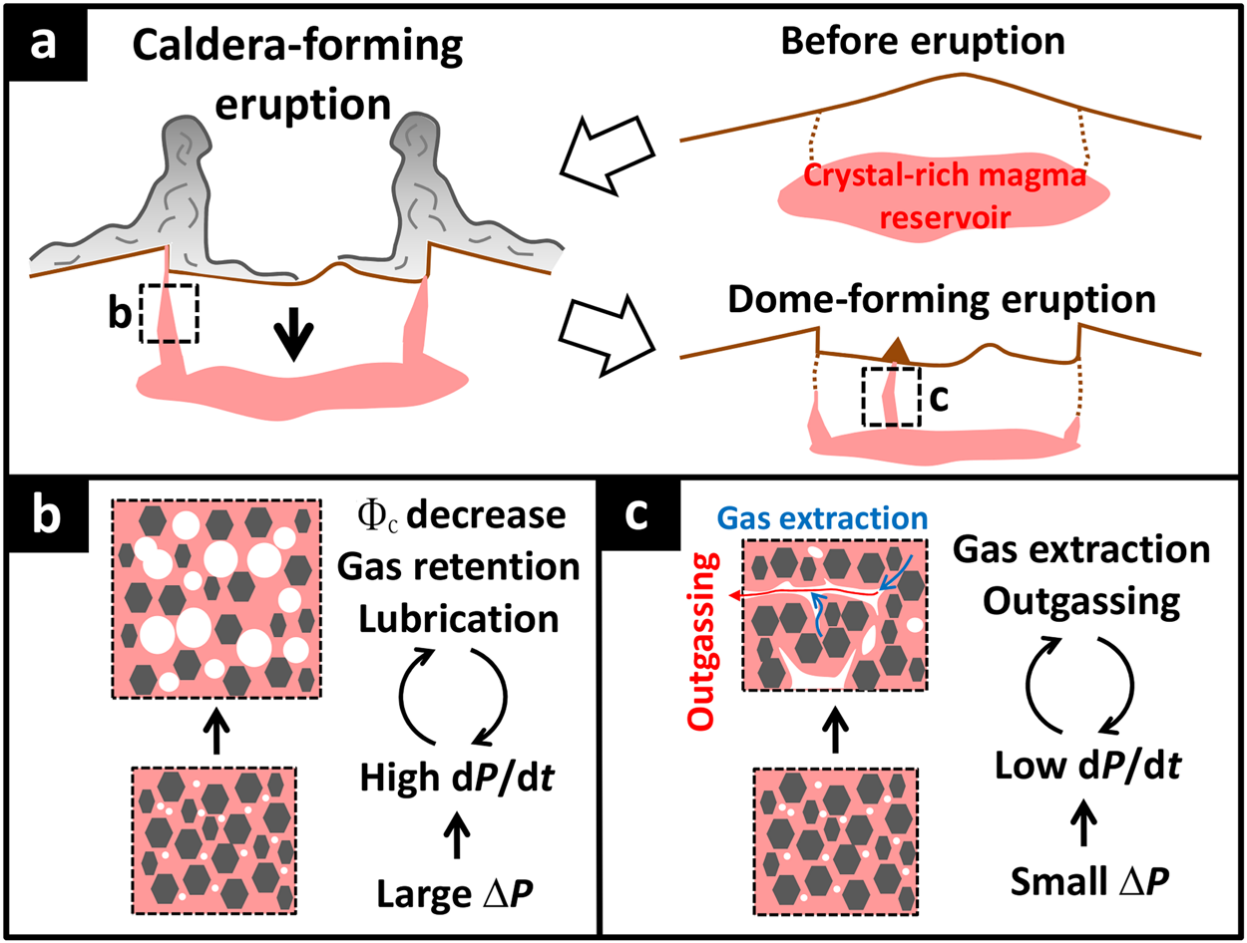
A schematic image of processes controlling the bifurcation of caldera-forming eruption and lava effusion. (**a**) Climatic ignimbrites and the post-caldera domes during the caldera-forming eruption. The climatic eruption is triggered by the roof collapse resulting in large overpressure and the post-caldera dome is caused by small overpressure in the reservoir. (**b**) Gas bubbles are retained in crystal-rich magma when the overpressure (∆*P*) in the reservoir is large and decompression rate (d*P*/d*t*) is high. In this case, d*P*/d*t* increases with the ascent of magma and crystal connectivity (Φ_c_) decreases. (**c**) Under low overpressure, d*P*/d*t* is low, which causes gas extraction and outgassing. This feedback loop causes non-explosive eruption.

Our observations from samples of the large Central Andean caldera-forming eruptions we have studied here extend this model to how crystal-rich magma ascends and erupts. The rapid decompression necessary to cause plug flow, gas retention and explosive eruption is the result of the subsiding roof of the magma reservoir acting like a plunger into the magma (stoping), forcing it out through excess pressure (Fig. 6a). The eruptive flux is also likely to increase as the roof subsides and vents expand^67^. Whereas shear deformation may occur in a thin annulus at the margins of the conduit as documented by minority sheared pumice, the predominance of non-sheared pumice and our new observations indicate that the main volume of the erupting mixture did not experience significant shear. The sheared annulus enables rapid ascent of the crystal-rich magma plug without outgassing, as shown in our experiments, driving explosive eruption (Fig. 6b). These processes are likely to be enhanced by the associated nonlinear increase of the partial molar volume of H_2_O fluid and H_2_O-CO_2_ fluid with decreasing pressure that can accelerate catastrophic collapse of the crystal networks once it starts to disrupt by the processes described above.

Eventually, as magmastatic, lithostatic, and isostatic equilibrium is reached between the subsiding block and the increasingly mushy magma at depth, subsidence is arrested and the pressure of the plunger is relieved. Subsequent eruptions are driven by local stress changes and pressurization associated with post-caldera magma dynamics (including recharge). These are commonly effusions of “remnant” magma^33,34,68^ and our investigation indicates that these magmas de-gas due to lower decompression rates and outgassing through the interaction between growing gas bubbles and crystal frameworks (Fig. 6c).

Our study helps understand explosive and effusive eruptions as part of a continuum linked by feedbacks between ascent rate, gas retention/loss and bubble/crystal framework interactions and can be applied to caldera-forming eruptions of crystal-rich silicic magmas in general.

## Methods

### Decompression experiments

All decompression experiments were conducted at Tohoku University, using a cold-seal pressure vessel connected to a syringe pump. In this study, we used a Rene41 pressure bomb, and the capsule was set in the bomb using a Ni filler rod. All experiments were conducted at a temperature of 800 °C. The samples were annealed at 100 MPa for ~20 h, and then decompressed to final pressure at various decompression rates. Using the syringe pump, the decompression rates were controlled at 8, 80, 320, and 3200 MPa h^−1^. Rapid decompression (~10 s) was also performed by opening a valve to release water (pressure medium). For the experiments, we used rhyolitic glass (reference material JR-1 from the Geological Survey of Japan) and 100 meshed corundum (Al_2_O_3_ crystal) (SIGMA-ALDRICH, Inc) as starting materials. The crystal-bearing samples were prepared by mixing the rhyolitic glass and corundum. We performed two series of the decompression experiments. In the first series (EX-I), samples were inserted into Au capsules, 5 mm in outer diameter, together with a known amount of water that corresponded to a melt water content of ~3 wt% at the experimental conditions of this study. After heating of the sample in the cold-seal pressure vessel, we rapidly decompressed hydrous rhyolitic melts with 50 vol% crystals from 100 to 40, 20, 15 and 10 MPa, by releasing water (pressure medium) via opening a valve, and from 100 to 20 MPa at a decompression rate of 8 MPa h^−1^. At 50 vol% crystal, roughly equant shape corundum crystals form the framework, resulting in high crystal connectivity. In the second series (EX-II), rhyolitic glass with 50 vol% crystal was sandwiched between crystal-free rhyolite glasses (JR-1) in the Au capsule with 5 mm outer diameter. Again, a known amount of water corresponding to 3 wt% melt water content was added to each capsule. These magmas were subjected to continuous decompression to 20 MPa with rates of 8, 80, 320, and 3200 MPa h^−1^ and rapid decompression. Two additional experiments were performed with the decompression rates of 8 MPa h^−1^ to investigate the effect of final pressure (20–40 MPa) on Φ_c_ and Φ_b_. All the experimental conditions and results are summarized in Table S1.

### X-ray CT

Microstructures of natural samples and run products were analyzed using a micro-X ray CT (ScanXmate-D180RSS270, Comscantecno Co, Ltd.) at Tohoku University. For CT analyses of natural samples, the sample core, ~20 mm in diameter, was first placed on a rotational stage and was subsequently rotated through 360° in 0.18° steps; its transmission image was obtained at each incremental step, resulting in 2000 projections. The acceleration voltage and current used to obtain the transmission images were 100–110 kV and 110–130 μA, respectively. Three-dimensional (3D) CT images with 8-bit resolution and a spatial resolution of 12–14 μm on one side of each voxel was reconstructed from the transmission images. For CT analyses of run products, the Au capsules were carefully removed. Owing to their fragility, some samples showed breakage during capsule removal (Fig. 3b). The acceleration voltage and current used to obtain the transmission images were 110 kV and 100–120 μA, respectively. The spatial resolution of the 3D CT images was 4–6 μm on one side of each voxel. To measure the volume fraction of gas bubbles and calculate bubble connectivity, defined as Φ_b_ = ϕ_m,b_/ϕ_b_, where ϕ_m,b_ and ϕ_b_ represent the volume of gas bubbles belonging to the largest bubble network and total bubble volume, respectively, the 8-bit (CT) images were binarized using a threshold at which the number of voxel shows the minimum from the CT value histogram. The volume fraction of gas bubbles (i.e., porosity) was determined from the binary images by counting the number of voxels belonging to gas bubbles and other phases based on a software package called “slice.” For this, the region of interest (ROI) in the crystal-bearing portion was manually selected. Total volumes of the ROI selected were 1.8 × 10^7^ − 4.9 × 10^7^ pixels. The volume fraction of crystals was also measured from the same 8-bit images. For the crystal measurements, we used a threshold, i.e., the 8-bit value between two peaks corresponding to crystal and glass. The connectivity of crystals, defined as Φ_c_ = ϕ_m,c_/ϕ_c_, where ϕ_m,c_ and ϕ_c_ represent the volume of crystals belonging to the largest crystal network and total crystal volume, respectively, was also calculated. Although we cannot identify the exact contact between crystals from CT images alone, we assume that crystals touch each other when the crystals cannot be separated in the CT images.

### Timescale estimate

We compared two timescales, pressure increase (*τ*_p_) and gas extraction (*τ*_ex_). For the extraction of gas-melt mixtures from crystal-rich zone to crystal-free zone, *τ*_ex_ can be described by the length scale (*l*) and velocity of the mixtures (*v*), which is given by the following equation^69^:

1 $$v=\frac{\kappa }{\eta }\frac{\nabla P}{\varphi },$$

where *κ*, *η*, and *ϕ* are the permeability, viscosity, and volume fraction of interstitial fluid phase, respectively, and ∇*P* is the pressure gradient. The value of *κ* is estimated from the relationship *α*^2^·*ϕ*^3^/[Α·(1 − *ϕ*)^2^] (ref.^70^), where *a* and *A* represent the grain radius (~50 μm) and a constant (~50 for grain size of ~0.5 mm, ref.^71^). For our experiments, *l* is ~1 mm. The viscosity (*η*) of the interstitial fluid, that is, the mixture of melt and gas bubbles, is assumed to be 1.26 × 10^6^ Pa s based on the relation of *η*/*η*_m_ = 0.1 at ~50 vol% porosity according to ref.^39^ and *η*_m_ = 1.26 × 10^7^ Pa s at 800 °C and 1.65 wt% water, corresponding to water saturation at 20 MPa, calculated based on the model of ref.^72^.

The timescale of bubble growth in viscous magma (*τ*_g_) is estimated using the ratio of magma viscosity (*η*_m_) to the degree of decompression (∆*P*) (ref.^73^). When melt viscosity is 1.26 × 10^7^ Pa s, the viscosity of magma with a crystal volume fraction of 50% is calculated to be 1.57 × 10^9^ Pa s based on the model of ref.^40^.

## Supplementary information

Supplementary Information

## Data Availability

All the data obtained in this study are available from the corresponding author upon request.
