# Supplementary material for: Caldera-forming eruptions of mushy magma modulated by feedbacks between ascent rate, gas retention/loss and bubble/crystal framework interaction
Source: Sci Rep. 2019 Nov 1;9:15845. doi: 10.1038/s41598-019-52272-9 (PMC6825124; doi:10.1038/s41598-019-52272-9)
Supplement: Supplementary file 1 — Supplementary Information [file 41598_2019_52272_MOESM1_ESM.pdf]

Supplementary Information of

Caldera-forming eruptions of mushy magma modulated by feedbacks between ascent rate, gas retention/loss and bubble/crystal framework interaction

Satoshi Okumura, Shanaka de Silva, Michihiko Nakamura, Osamu Sasaki

Supplementary Figure

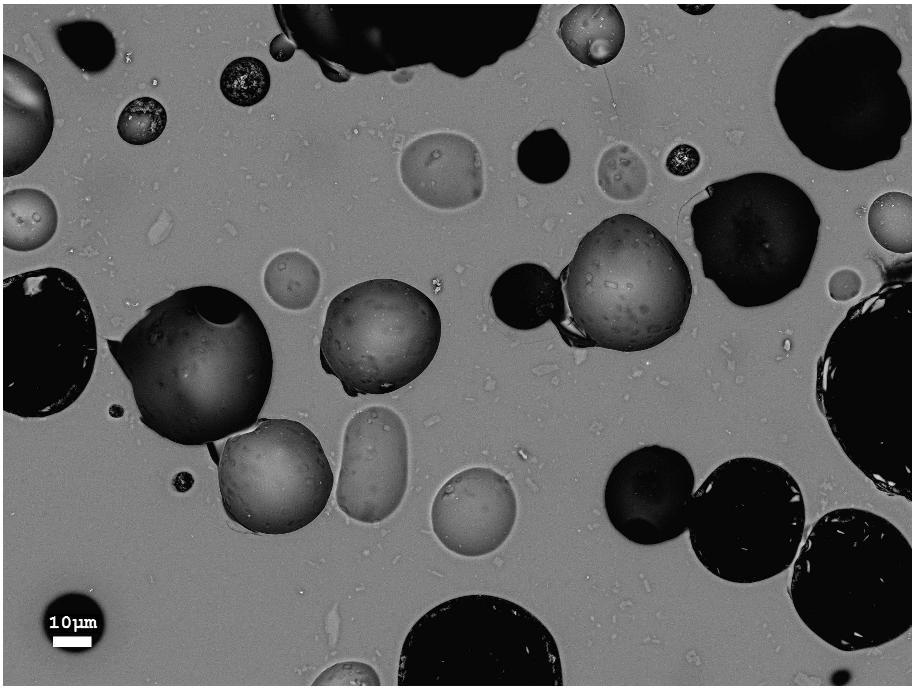

Figure S-1. A backscattered electron image of crystal-free melt part in a run product with a decompression rate of 8 MPa/h (EXII-50-8). Grey portion is glass part; small light grey particles found in the glass part (< 10 µm) are plagioclase microlite.

Supplementary Table

Table S1 | Experimental conditions and results

Run#	Crystal fraction [vol%]	Final P [MPa]	Decompression rate [MPa/h]	Bulk vesicularity[a] [vol%]	Bulk crystallinity[b] [vol%]	Bubble connectivity	Crystal connectivity
EX-I-100	50	100	RD (28800)[c]	3	38	0.15	0.99
EX-I-100	50	100	RD (28800)[c]	1	46	0.13	0.99
EX-I-40-RP	50	40	RD (28800)[c]	9 (8)[d]	39	0.12	0.98
EX-I-20-RP	50	20	RD (28800)[c]	28 (28)	33	0.83	0.98
EX-I-15-RP	50	15	RD (28800)[c]	30 (39)	33	0.96	0.91
EX-I-10-RP	50	10	RD (28800)[c]	31 (52)	27	0.93	0.54
EX-I-20-8	50	20	8	12 (18)	31	0.28	0.99
EX-II-20-RP	50	20	RD (28800)[c]	38	22	0.87	0.82
EX-II-20-RP-2	50	20	RD (28800)[c]	26	32	0.94	0.78
EX-II-20-3200	50	20	3200	38	32	0.99	0.63
EX-II-20-320	50	20	320	32	36	0.98	0.95
EX-II-20-80	50	20	80	13	34	0.17	0.97
EX-II-20-8	50	20	8	6	56	0.03	1.00
EX-II-20-8-2	50	20	8	12	51	0.04	1.00
EX-II-30-8	50	30	8	14	47	0.14	1.00
EX-II-40-8	50	40	8	9	42	0.03	0.99

[a] The bulk vesicularity represents the proportion of pore to solid phase (glass + crystal).

[b] The bulk crystallinity represents the volume fraction of crystals on bulk sample.

[c] Decompression rate for rapid decompression (RD) is estimated assuming decompression time of 10 s, resulting in 28800 MPa/h.

[d] The values in parentheses represent the theoretical bulk vesicularities.
